# Right atrial rupture due to cardiac angiosarcoma without haemodynamic collapse: a case report of successful multidisciplinary management

**DOI:** 10.1093/ehjcr/ytag523

**Published:** 2026-07-11

**Authors:** Toshiki Fukuda, Yoko Horibata, Mamoru Ito, Takeshi Iwasaki, Junjiroh Koyama

**Affiliations:** Division of Cardiology, Cardiovascular Center, Saiseikai Kumamoto Hospital, 5-3-1 Chikami, Minami-ku, Kumamoto 861-4193, Japan; Division of Cardiology, Cardiovascular Center, Saiseikai Kumamoto Hospital, 5-3-1 Chikami, Minami-ku, Kumamoto 861-4193, Japan; Department of Hematology, Oncology and Cardiovascular Medicine, Kyushu University Hospital, 3-1-1 Maidashi, Higashi-ku, Fukuoka 812-8582, Japan; Department of Anatomic Pathology, Graduate School of Medical Sciences, Kyushu University, 3-1-1 Maidashi, Higashi-ku, Fukuoka 812-8582, Japan; Division of Cardiology, Cardiovascular Center, Saiseikai Kumamoto Hospital, 5-3-1 Chikami, Minami-ku, Kumamoto 861-4193, Japan

**Keywords:** Cardiac angiosarcoma, Atrial wall rupture, Surgical resection, Chemotherapy, Case report

## Abstract

**Background:**

Malignant tumours account for approximately 20%–30% of primary cardiac tumours, and reports remain rare. Cardiac angiosarcoma is a representative type, but prognosis is extremely poor: the median survival is about 4 months in unresectable cases and 14 months after complete resection [Patel SD, Peterson A, Bartczak A, Lee S, Chojnowski S, Gajewski P, *et al*. *Med Sci Monit* 2014;**20**:103–9].

Approximately 29% of angiosarcomas present with metastasis at diagnosis, and the presence of pericardial effusion suggests pericardial invasion. Evidence regarding chemotherapy remains limited, and no standardized treatment has been established for metastatic primary cardiac tumours.

**Case summary:**

We report a case of cardiac angiosarcoma in a 74-year-old man. At the time of presentation, masses were detected in the right atrium and the apex of the left ventricle. The right atrial tumour extended widely along the atrial wall and partially formed fistulous tracts, resulting in direct communication of blood flow from the right atrium into the pericardial cavity. Surgical tumour resection with atrial wall reconstruction was performed, and chemotherapy was initiated following histopathological confirmation.

**Discussion:**

Due to their poor prognosis, cardiac tumours are often managed with palliative care. This case highlights two important points: first, cardiac angiosarcoma can cause atrial wall destruction and rupture, leading to direct communication between the atrium and pericardial space; second, even in cases of primary cardiac angiosarcoma with pulmonary metastases, appropriate cardiac surgery combined with chemotherapy may improve patient outcomes.

Learning points

- In cardiac angiosarcoma, atrial wall rupture may lead to direct communication between the atrium and pericardial cavity.
- When the tumour does not form a discrete mass, surgical resection combined with pathological confirmation enables initiation of chemotherapy.

## Introduction

Angiosarcoma is a malignant tumour of vascular endothelial origin. It most commonly arises in the soft tissues of the head, neck, and extremities, but cases in the heart and liver have also been reported.^1^ Angiosarcoma accounts for less than 2% of all soft tissue sarcomas,^2,3^ making it extremely rare. Among primary cardiac tumours, approximately 25% are malignant, of which about one-third are angiosarcomas. They typically occur in adults, with a mean age of 40 years and a male predominance of 2:1.^4^

Primary cardiac angiosarcoma accounts for 4%–7% of angiosarcomas.^1^ Clinically, patients usually present with intracardiac obstruction, embolism, or arrhythmias, and the tumour shows a predilection for the right atrium. Complete surgical resection remains the gold standard of treatment; however, even in patients without metastasis, the median survival after diagnosis is reported to be only 9–12 months.^5^ While some reports have suggested chemotherapy to be beneficial, most are small series due to the rarity of the disease, and no standardized regimen has been established.

## Summary figure

**Figure:**
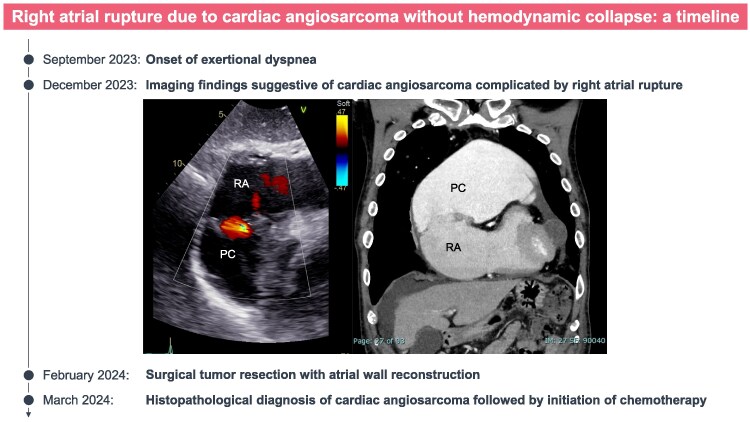


## Case presentation

A 74-year-old man with chronic atrial fibrillation but no other significant comorbidities presented to a local clinic in September 2023 with exertional dyspnoea. Non-contrast chest computed tomography (CT) revealed multiple pulmonary nodules, raising suspicion of metastatic lung disease, but no primary lesion was identified. A small amount of pericardial effusion was already noted at that time, and contrast-enhanced CT was not performed. At follow-up in December, progression of pericardial effusion was detected, and he was referred to the cardiology department.

On presentation, there were no symptoms suggestive of cardiac tamponade. Vital signs were stable, and electrocardiography demonstrated atrial fibrillation. Bilateral leg oedema indicated right heart failure, but no left heart failure was found.

Previously noted pulmonary nodules were again observed on non-contrast CT (*Figure 1*), while contrast-enhanced CT demonstrated progression of pericardial effusion with contrast leakage from the right atrium into the pericardial cavity (*Figure 2*). The effusion was mainly localized anterior to the right atrium, sparing the ventricles. A small amount of pleural effusion and mediastinal lymphadenopathy were also detected. No significant coronary artery stenosis was found.

**Figure 1:**
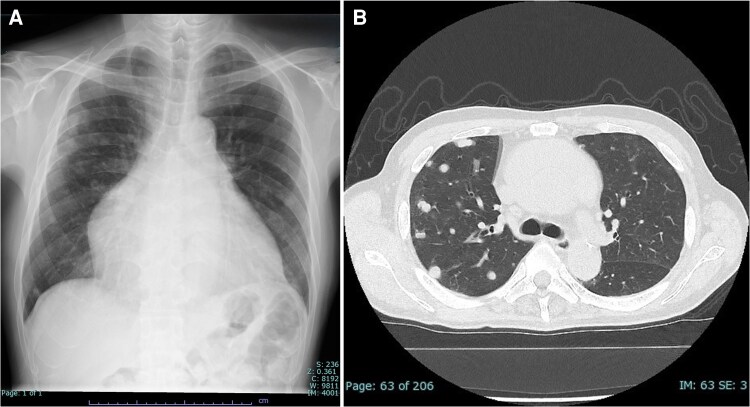
Chest X-ray and computed tomography. Chest X-ray revealed cardiomegaly *(A)*, and computed tomography (CT) demonstrated multiple nodular opacities in both lungs *(B)*.

**Figure 2:**
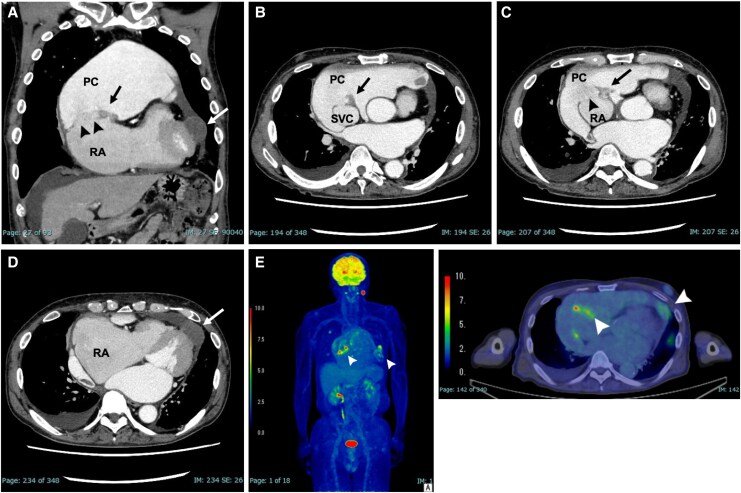
Contrast-enhanced CT and FDG-PET/CT. Contrast-enhanced CT demonstrated a mass lesion in the upper portion of the right atrial free wall (black arrows; *A*, *B*, and *C*) with extravasation of contrast medium from the surrounding area (black arrow heads; *A* and *C*). A 38-mm mass was also identified at the left ventricular apex (white arrow; *D*). FDG-PET/CT revealed hypermetabolic activity at the superior vena cava–right atrial junction, the right atrial free wall, and the left ventricular apex (SUVmax 18.3, 15.6, and 6.2, respectively) (white arrow heads; *E*). PC, pericardial cavity; RA, right atrium; SVC, superior vena cava.

Cardiac magnetic resonance imaging (CMR) revealed a 20-mm lesion protruding from the superior free wall of the right atrium and a 30-mm lesion at the left ventricular apex, both of which were hypointense on T1- and T2-weighted imaging, showed heterogeneous enhancement during the early contrast phase, and demonstrated diffuse enhancement of the entire mass on late gadolinium imaging (*Figure 3*).

**Figure 3:**
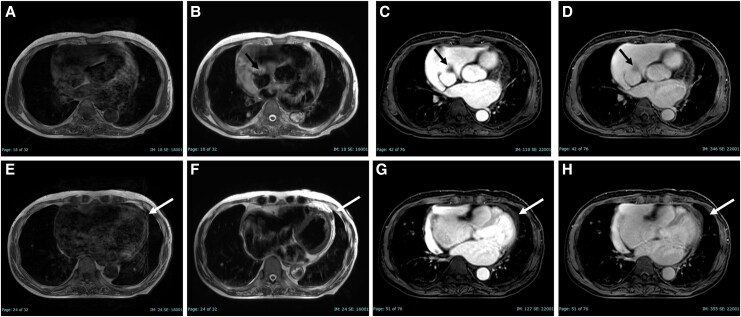
Cardiac magnetic resonance imaging (CMR). A low-signal nodule, approximately 20 mm in size, protruding outward from the upper portion of the right atrial free wall was observed (black arrows; *A*, *B*, *C*, and *D*). The lesion exhibited heterogeneous enhancement in the early contrast phase and homogeneous enhancement in the delayed phase. Extravasation of contrast medium from the anterior aspect of the right atrium was also noted. In addition, a 38-mm mass was identified at the left ventricular apex (white arrows; *E*, *F*, *G*, and *H*). Among these MRI images, *A* and *E* show T1-weighted images (T1WI), *B* and *F* show T2-weighted images (T2WI), *C* and *G* show the early phase (early gadolinium enhancement, EGE), whereas *D* and *H* show the late phase (late gadolinium enhancement, LGE).

Transthoracic echocardiography demonstrated a markedly enlarged right atrium with pericardial effusion. Multiple fistulous tracts were identified from the superior vena cava–right atrial junction to the pericardial cavity (see Supplementary material online, *Video S1*). Left ventricular ejection fraction was preserved; severe tricuspid regurgitation was noted. There was no haemodynamic compromise; inferior vena cava respiratory variation was preserved, without right ventricular diastolic collapse. Transoesophageal echocardiography confirmed anterior right atrial wall destruction with multiple fistulas and revealed a left ventricular apical mass.

Laboratory data showed BNP 122.5 pg/mL and normal renal and hepatic function. Fluorodeoxyglucose-positron emission tomography/CT (FDG-PET/CT) demonstrated increased uptake in both the right atrial and left ventricular apex lesions, as well as in pulmonary nodules, thoracic vertebrae, and ribs (*Figure 2*).

We considered several methods for obtaining a histopathological diagnosis. However, the right atrial lesion lacked a distinct mass, and the left ventricular apical lesion did not reach the endocardial surface; thus, catheter-based biopsy was deemed inappropriate. Because of blood flow from the right atrium into the pericardial cavity, pericardiocentesis from the apical region for cytological examination was also considered unsafe.

A multidisciplinary team discussion involving cardiologists, cardiac surgeon, respiratory physicians, medical oncologists, and radiologists were held to determine the treatment strategy. Transbronchial lung biopsy, CT-guided lung biopsy, and open lung biopsy were all considered unsuitable for tissue diagnosis. Therefore, cardiac surgery was selected for both diagnosis and treatment. In February 2024, the patient underwent median sternotomy under cardiopulmonary bypass. The right atrial tumour extending to the superior vena cava was partially resected, and the atrial wall was reconstructed using an equine pericardial patch. The left ventricular apical tumour was excised *en bloc* with pericardium.

Postoperative recovery was uneventful. Histopathological examination confirmed cardiac angiosarcoma (*Figure 4*). Weekly paclitaxel (150 mg/body) chemotherapy was initiated in March according to the ANGIOTAX protocol. Both the right atrial and left ventricular apex lesions had the same histological diagnosis. The patient tolerated chemotherapy well without significant adverse effects and was discharged home.

**Figure 4:**
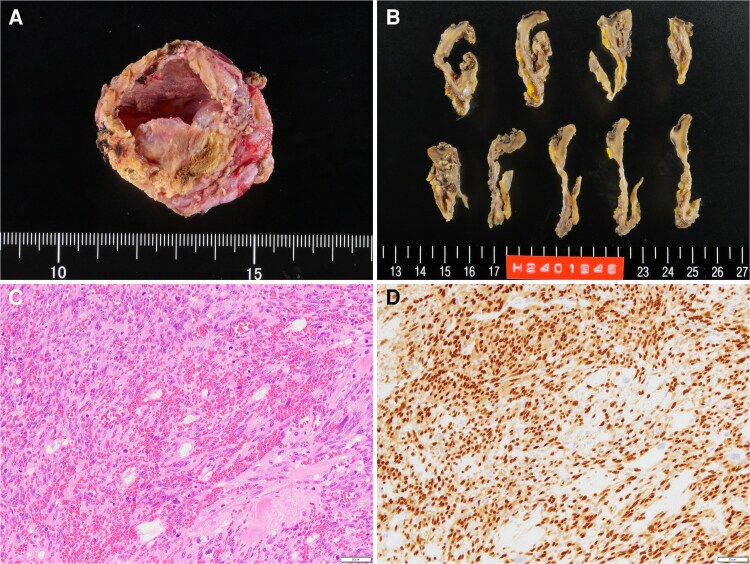
Pathology. (*A*) Macroscopic pathological view of the right atrial fistula. (*B*) Macroscopic pathological view of the left ventricular tumour. (*C*) Haematoxylin and eosin (HE) staining (×20): Right atrial tissue shows a proliferation of atypical spindle cells arranged in fascicular pattern, accompanied by extravascular red blood cells. Left ventricular tissue shows inflamed granulation tissue with foreign body resection. Some foci of proliferated atypical spindle cells are observed at the surface of endocardium. (*D*) ERG immunohistochemical staining (×20): Immunohistochemically, the atypical spindle cells are positive for CD31, CD34, ERG, and focally Desmin and p16, but negative for AE1/AE3 and mdm2.

Follow-up CT demonstrated closure of the atrial–pericardial fistula; however, small residual enhancing foci were observed around the right atrial wall, suggesting residual tumour.

Chemotherapy was continued, and at one year, he remained alive with stable disease despite persistent right heart failure.

## Discussion

We report a case of primary cardiac angiosarcoma complicated by right atrial wall destruction and rupture. Malignancy accounts for approximately 13%–23% of pericardial effusions.^6^ The mechanisms of malignant pericardial effusion include direct pericardial invasion, lymphatic spread to mediastinal lymph nodes, and haematogenous metastasis. In this case, destruction of the right atrial wall by angiosarcoma resulted in direct blood flow from the atrium into the pericardial cavity, causing atrial rupture.

Approximately 90% of primary cardiac angiosarcomas arise in the right atrium.^7^ Reports of cardiac angiosarcoma complicated by atrial wall rupture are extremely rare.^8,9^ Pseudoaneurysm formation has also been described.^8^ The likely mechanisms include destruction of atrial wall structure by direct tumour infiltration, along with tissue fragility caused by tumour necrosis.

Notably, despite right atrial rupture, our patient did not develop tamponade or haemodynamic instability. In contrast, left ventricular rupture generally leads to shock or cardiac arrest due to acute circulatory collapse. In this case, however, only exertional dyspnoea was observed, and vital signs remained stable. Previous reports of pericardial effusion associated with cardiac angiosarcoma have described the absence of tamponade.^9^ Possible explanations include relatively low right atrial pressure and gradual blood leakage, allowing slow progression without abrupt deterioration, as well as the localized distribution of pericardial effusion that did not surround the ventricles. The absence of contrast flow into the periventricular space may have been due to thrombus formation or pericardial adhesions.

Histological confirmation, surgical resection, and chemotherapy based on histopathological diagnosis are generally considered the standard of care. However, obtaining a tissue diagnosis was challenging in this case. Even in haemorrhagic pericardial effusion caused by pericardial infiltration of angiosarcoma, the sensitivity of cytology is low.^10^ Pericardiocentesis was considered but deemed unsafe due to direct atrial–pericardial fistulous communication, which carried a high risk of haemodynamic collapse.

The pulmonary metastases were located peripherally and not accessible by transbronchial biopsy. Because the cardiac tumour lacked a discrete mass, catheter-based biopsy was also considered inappropriate. Ultimately, surgical resection was chosen to achieve both tissue diagnosis and life-saving treatment. Histopathology confirmed cardiac angiosarcoma, and weekly paclitaxel was administered according to the ANGIOTAX study.^11^

This case highlights that even in the presence of right atrial rupture due to cardiac angiosarcoma with pulmonary metastases, aggressive multidisciplinary treatment combining surgery and chemotherapy can contribute to improved survival.

## Conclusion

We report a rare case of cardiac angiosarcoma in which right atrial wall destruction led to direct communication between the atrium and pericardial cavity. Following multidisciplinary discussion, the patient underwent surgical tumour resection with atrial wall reconstruction, followed by chemotherapy after histopathological confirmation.

## Supplementary Material

ytag523_Supplementary_Data

## Data Availability

The data underlying this article are available within the article.

## Lead author biography

I graduated from Nagasaki University and received the MD degree 2019. I had cardiology training in Department of Cardiology, Saiseikai Kumamoto Hospital.

## Notes

**Consent:** Written informed consent was obtained from the patient for publication of this case report and any accompanying images, in accordance with COPE guidelines.
