# Supplementary figures and images for: Right atrial rupture due to cardiac angiosarcoma without haemodynamic collapse: a case report of successful multidisciplinary management
Source: Eur Heart J Case Rep. 2026 Jul 11;10(7):ytag523. doi: 10.1093/ehjcr/ytag523 (PMC13422633; doi:10.1093/ehjcr/ytag523)

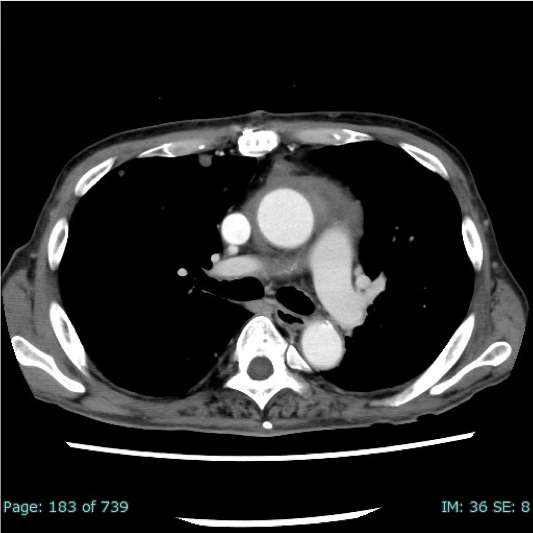

Supplement: ytag523_Supplementary_Data [file ytag523_supplementary_data.zip › CT after surgery A.jpg]

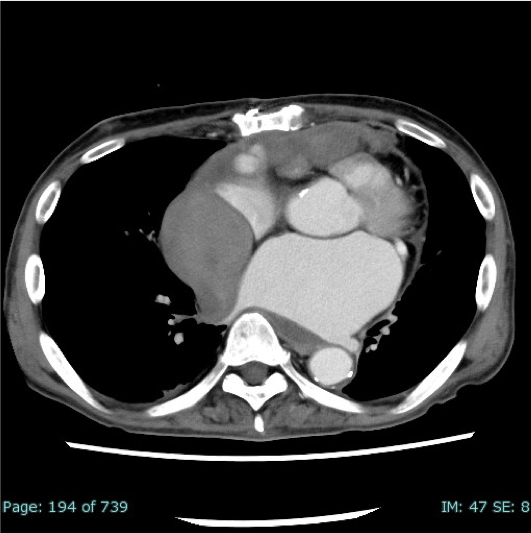

Supplement: ytag523_Supplementary_Data [file ytag523_supplementary_data.zip › CT after surgery B.jpg]

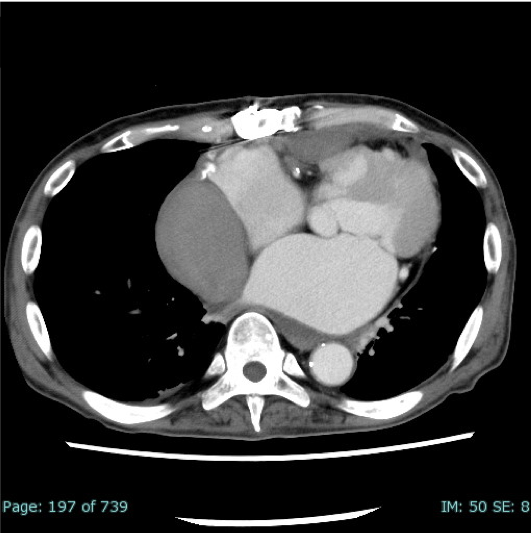

Supplement: ytag523_Supplementary_Data [file ytag523_supplementary_data.zip › CT after surgery C.jpg]

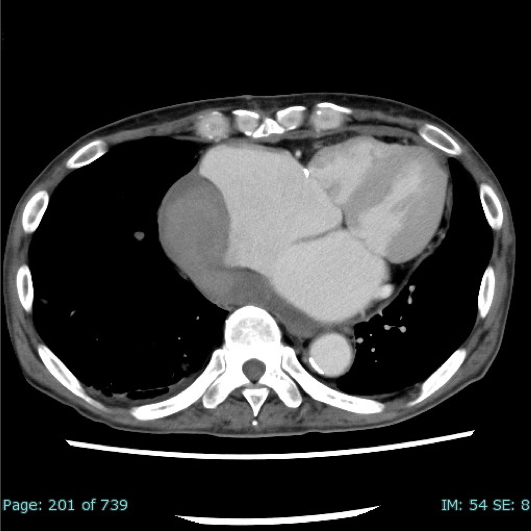

Supplement: ytag523_Supplementary_Data [file ytag523_supplementary_data.zip › CT after surgery D.jpg]
